# The Influence of MgH_2_ on the Assessment of Electrochemical Data to Predict the Degradation Rate of Mg and Mg Alloys

**DOI:** 10.3390/ijms150711456

**Published:** 2014-06-26

**Authors:** Wolf-Dieter Mueller, Helga Hornberger

**Affiliations:** 1 Biomaterial Research CC3, Dental School Charité Universitaetsmedizin Berlin, Assmannshauserstrasse 4-6, Berlin 14197, Germany; 2 Department Material Science and Technology EB13, Technische Universität Berlin, Strasse des 17. Juni 13, Berlin 10623, Germany; E-Mail: helga.hornberger@tu-berlin.de

**Keywords:** magnesium, magnesium alloys, magnesium hydride, corrosion, degradation

## Abstract

Mg and Mg alloys are becoming more and more of interest for several applications. In the case of biomaterial applications, a special interest exists due to the fact that a predictable degradation should be given. Various investigations were made to characterize and predict the corrosion behavior *in vitro* and *in vivo*. Mostly, the simple oxidation of Mg to Mg^2+^ ions connected with adequate hydrogen development is assumed, and the negative difference effect (NDE) is attributed to various mechanisms and electrochemical results. The aim of this paper is to compare the different views on the corrosion pathway of Mg or Mg alloys and to present a neglected pathway based on thermodynamic data as a guideline for possible reactions combined with experimental observations of a delay of visible hydrogen evolution during cyclic voltammetry. Various reaction pathways are considered and discussed to explain these results, like the stability of the Mg^+^ intermediate state, the stability of MgH_2_ and the role of hydrogen overpotential. Finally, the impact of MgH_2_ formation is shown as an appropriate base for the prediction of the degradation behavior and calculation of the corrosion rate of Mg and Mg alloys.

## 1. Introduction

Magnesium (Mg) and its alloys are applied in different fields, such as the automotive and aircraft industries, as well as biomedical applications [1]. The advantage of magnesium or Mg alloys for most fields of application is the high strength-density ratio. Depending on the field of application, as possible candidates for biologically biodegradable biomaterials, the corrosion stability or the degradation rate is of special interest. In aqueous electrolytes, the corrosion of magnesium and Mg alloys can be easily assessed by the immersion test, by weight loss measurements, combined with the eudiometric volume measurement of hydrogen, based on the well-known reaction (Equation (1)), according to Atrens *et al.* [2].

*Mg* + *H*_2_*O* ⇒ *Mg(OH)*_2_ + *H*_2_ (1)

Another possibility to assess the corrosion behavior is given by the application of electrochemical measurement techniques, such as open circuit potential (OCP) measurements, cyclic voltammetry (CV) and electrochemical impedance spectroscopy (EIS) [3,4,5,6,7,8,9]. However, using electrochemical methods one problem appears: the hydrogen evolution at anodic polarization during the measurements. This fact complicates the assessment of current (*I*)–voltage (*E*) curves for estimating the corrosion rate based on the exchange current density (*i*_corr_) using the Tafel kinetic or the polarization resistance method. Electrochemical measurements are very useful for the testing of new alloys or treatments to compare the corrosion behavior in relatively short time periods and small sample batches compared with immersion test, especially by the use of the mini-cell system [10].

The controlled degradation of Mg and Mg alloys by the adjustment of the composition and microstructures or protecting layers and coatings requires a better understanding of the electrochemical processes that are responsible for corrosion [11,12,13,14,15]. The discrepancy between *in vitro* and *in vivo* degradation rates [16] is another reason for the necessity to discuss the different reaction pathways for Mg and Mg alloys and to start the interpretation of electrochemical data again, to achieve the reliable prediction of degradation rates. The aim of this paper is to present the hypothesis that Mg hydride is forming a stable intermediate reaction product during the corrosion, respectively degradation process of magnesium and, based on this hypothesis, to explain the resulting measured data. Furthermore, based on a summary of the data from the literature and the first experimental approach, the indication for the existence of Mg hydride as a stable intermediate will be given. With this new approach, it might be possible to develop the estimation of true corrosion rates.

## 2. Literature Survey

It was found experimentally that the directly measured hydrogen evolution rate (HER) increased with increasing potential [6,8,17], whereas the hydrogen evolution calculated from corrosion density measurements should decrease in the anodic direction. This effect is defined as the negative difference effect (NDE). Perrault [3] presented three different models of the Mg corrosion mechanism in aqueous electrolyte to explain NDE:

(1) NDE is attributed to the breakdown of a partially protective film on the magnesium surface during anodic dissolution. The formation of this layer is based on the creation of the Mg(OH)_2_ layer, which covers the surface and which changes with time to the MgO layer, liberating a molecule of water, according to Equation (2).
  *Mg*^2+^ + 2*OH*^−^ ⇒ *Mg(OH)*_2_ ⇒ *MgO* + *H*_2_*O* (2)
(2) Mg reacts in two steps, creating a monovalent Mg^+^ ion, which, in a second step, oxidizes to Mg^2+^, where some site steps are discussed for the creation of hydrogen as the final product; see Equations (3) and (4).

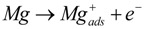
(3)

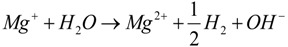
(4)
(3) The first layer created on the surface of Mg is a MgH_2_ layer, which, in a following reaction step with (OH)^−^ ions, liberates hydrogen and creates Mg(OH)_2_; see Equations (5) and (6).
  *Mg* + 2*e*^−^ + 2*H*_2_*O* → *MgH*_2_ + 2*OH*^−^ (5)
  *MgH*_2_ + 2*H*_2_*O* → *Mg(OH)*_2_ + 2*H*_2_ (6)

Mechanism 1 is supported by studies of the partially protective film, which was detected as a MgO/Mg(OH)_2_ layer by X-ray photoelectron spectroscopy (XPS) [18]. Santamaria *et al.* [18] additionally studied the film directly in organic and aqueous solutions using photocurrent spectroscopy (PCS), which did not show the composition, but the thickness of the layer.

Song *et al.* [8] discussed those mechanisms thoroughly and developed Mechanism 1 by adapting the NDE definition. Mechanism 2 was not satisfying, due to the low stability, and Mechanism 3 appeared be contradictory, as Mg hydride is not stable in water. EIS studies in chloride and sulfate solutions by Song *et al.* [8] showed that a partially protective surface film played an important role in the dissolution of magnesium, and furthermore, their data correlated with the involvement of the adsorbed intermediate species Mg^+^ at the film-free parts of the surface. They argued that the magnesium hydride model cannot explain the increase of hydrogen evolution with increasing anodic potential when there is no film on a magnesium surface. Furthermore, magnesium hydride is not stable and cannot exist in an acid solution. In the meantime, Mg hydride was detected by ToF-SIMS in the surface film, which is formed on magnesium by immersion for 2 min, but due to the low concentration, the conclusion was that MgH_2_ plays a minor role [19].

Nowadays, Mechanism 2 is often used as an approach to explain the NDE with the existence of Mg^+^ ions as stable intermediates [2,5,6,20], which produce hydrogen, according to Equation (4) [21]. However, Mg^+^ might be adsorbed or solved in the vicinity of the surface and not able to take part in the electron transfer connected to the bulk. Furthermore, the formation of Mg^+^ did not explain the discrepancy between measured and calculated HER in the study of Abidin *et al.* [20]. This possibly explains the findings of Dietzel *et al.* [22] and Weber *et al.* [6], where the HER in pits is much higher than at the surface. Lee *et al.* [23] tried to interpret the NDE by a chunk breakage and the creation of a protective film at the surface, but they did not explain how the surface film is chemically composed. Beside the difficulties in explaining NDE, the calculation of the corrosion rates of Mg based on *I* *vs.* *E* curves using the Tafel kinetic is not reliable. Abidin *et al.* [20] stated a difference between instantaneously or short time and steady state or long time measurements and mentioned that the application of the Tafel extrapolation might have measured a valid corrosion rate for the moment. In contrast, Thomaz *et al.* [24] and Nam *et al.* [25] said that the extrapolation of the cathodic curve leads to erroneous results.

## 3. Results

### 3.1. Open Circuit Potential (OCP) Measurements

**Figure 1:**
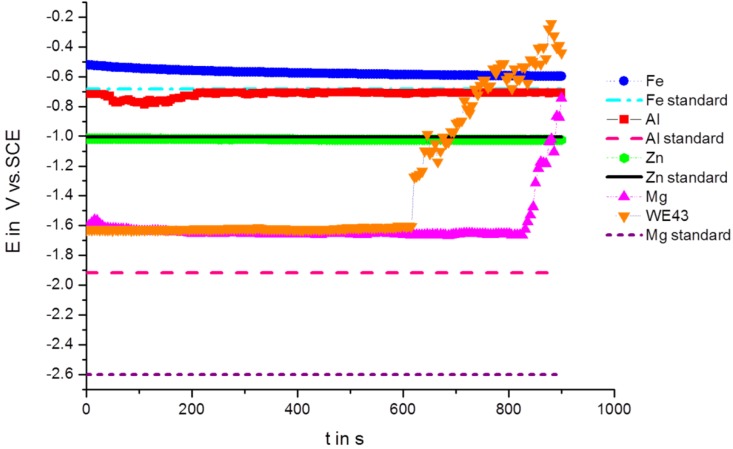
Open circuit potentials (OCPs) *vs.* time in Ringer solution for Mg, WE43, Fe, Al and Zn.

Figure 1 shows the OCP measurements over time at room temperature, the measurements were performed *vs.* a saturated calomel electrode (SCE). The OCPs are collected in Table 1. Figure 1 shows clearly that after 600 s for WE43 and after 800 s for pure Mg, the corrosion potential is dramatically shifted in the anodic direction. Observed via stereomicroscopy, the samples of Mg and WE43 reacted as soon as they came into contact with the electrolyte, and very small bubbles developed and adhered to the surface. However, the high increase of OCP was connected with strong hydrogen evolution, and the bubbles became large and took off from the surface. Additional measurements with increased time periods showed that the OCPs decreased again with time.

**Table 1:** Open circuit potentials after 15 min in Ringer’s solution. SCE, saturated calomel electrode.

Material	*E*_OCP_ (V *vs.* SCE) up to Break	*E*_OCP_ (V *vs.* SCE) after 15 min
Al pure	–	−0.709
Fe pure	–	−0.597
Zn pure	–	−1.025
Mg pure	−1.664	−0.744
WE43	−1.601	−0.44

### 3.2. Polarization Using a Scan Rate of 100 mV/s

**Figure 2:**
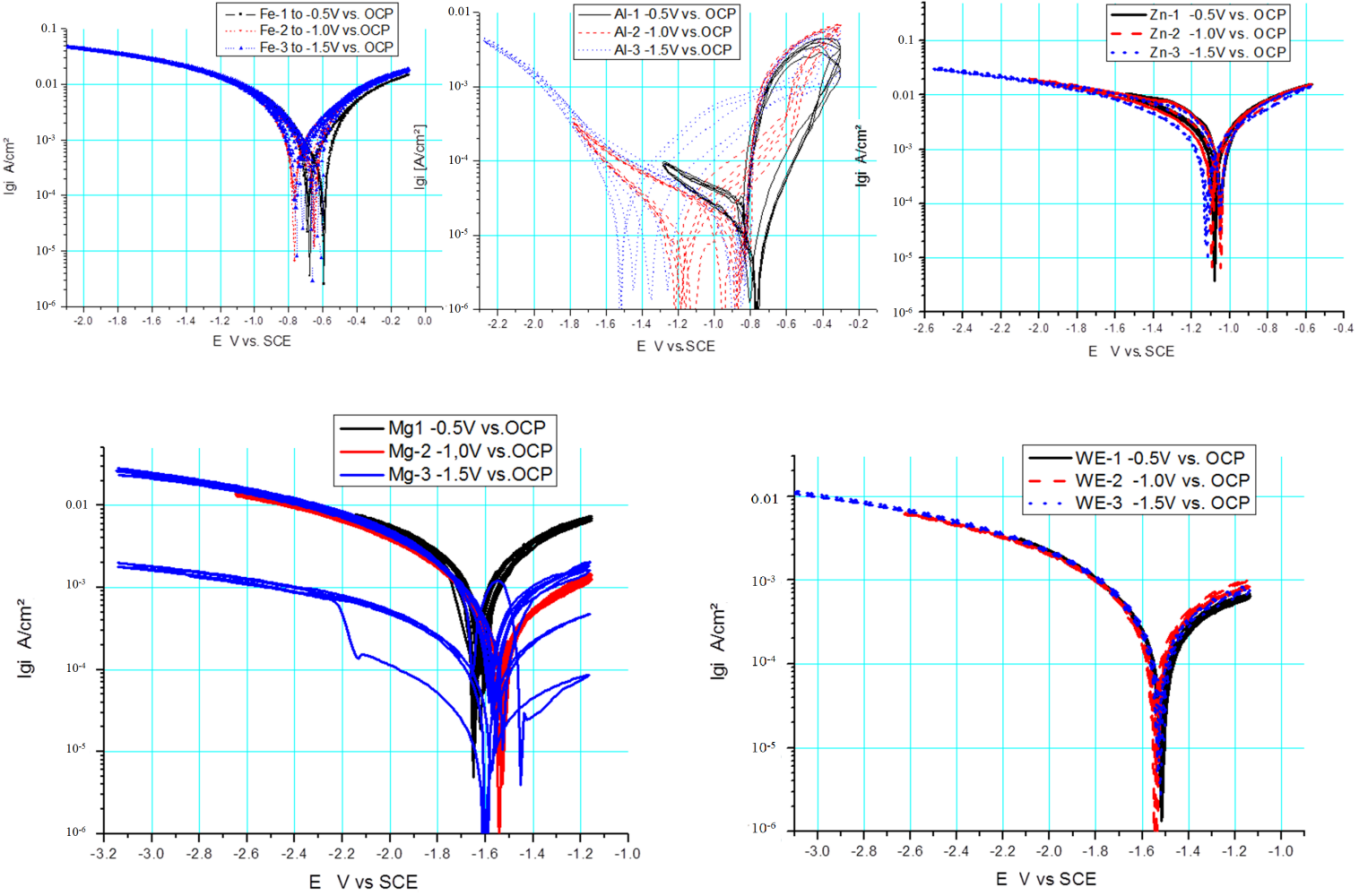
Cyclic current (*I*) *vs.* voltage (*E*) curves of different elements (Zn, Fe, Mg) and alloy WE43 in Ringer solution; scan rate: 100 mV/s.

*I* *vs.* *E* curves were measured with a scan rate of 100 mV/s at room temperature in cyclic voltammetry. The results of various metallic materials are presented as voltammograms in a semi-logarithmic scale in Figure 2. Usually, linear diagrams are used for cyclic voltammetry, but the logarithmic scale was chosen for a better view and the observation of changes. The voltammetry starts with the first five cycles (forward and backward scan) at a cathodic threshold of −0.5 V from OCP, marked as black lines (Figure 2), then proceeding with the second five cycles at a cathodic threshold of −1 V from OCP, marked as red lines, and the last five cycles were performed at a cathodic threshold of −1.5 V, marked as blue lines, whereas the anodic threshold was kept at +0.5 V from OCP for all cycles. The voltammograms show characteristic shapes for any of the materials. Zn shows a very reproducible behavior, including the corrosion potential, which is not even really dependent on the cathodic threshold potential. Fe shows a similar behavior with slight shifts of the corrosion potential for the forward and backward scan in relation to the extension of the cathodic threshold potential. In contrast, Al shows a significant dependency on the cathodic threshold potential for the forward scan, but not for the backward scan. A typical shape for pitting corrosion can be observed at the end of the anodic branch.

Mg shows different behavior for the forward scan in dependency of the cathodic threshold potential. During the first five cycles, the polarization takes place in the vicinity of the corrosion potential (OCP ± 0.5 V) and generates a very reproducible cyclic voltammogram regarding the forward and backward scan. With the extension of the cathodic threshold potential of −1 V from OCP, a shift of the corrosion potential in the anodic direction can be observed for the forward scan, and additionally, the anodic part shows reduced current densities. The reduction of current densities was combined with the enhanced evolution of bubbles adhered to the surface, causing a reduction of the electrochemically active and free surface. When the cathodic threshold potential finally reached −1.5 V from OCP and the final five cycles were performed, after two cycles, a strong hydrogen evolution started under anodic polarization during the forward scan, and the creation of large bubbles at the surface blocked the active part and reduced the current flow, even in the cathodic branch of the following cycle, which is marked with arrows in Figure 2. In contrast, WE43 does not show any of such variations as pure Mg. WE43 appears to have a very reproducible behavior, independent of the cathodic threshold potential; only a slight reduction of the current density in the anodic branch of the cyclic voltammogram can be seen in Figure 2. In both cases, Mg and WE43, an increase of the gas evolution and take-off of bubbles could be observed towards the anodic threshold potential, which means a breakdown of any partially protective film. For WE43, the gas evolution was “homogeneously” distributed onto the surface of the measurement area in contrast to pure Mg. On the surface of pure Mg, the gas bubbles accumulated quickly into one large bubble, as the partial pressure of the gas is higher than on the WE43 surface and could interrupt the current flow. After the take-off of the gas bubble and the compensation of that overpressure, the current flows at the same height as before, without any delay.

### 3.3. Comparison of Scan Rates at 100 and 10 mV/s

**Figure 3:**
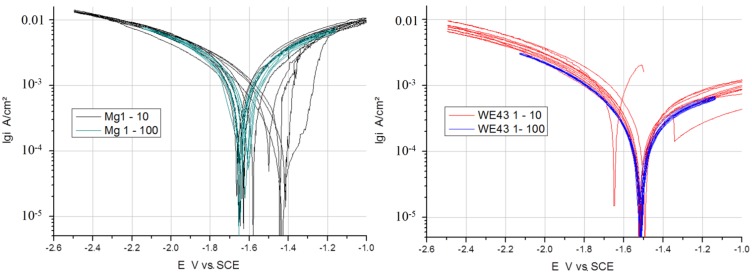
Comparison of *I* *vs.* *E* curves from Mg (**left**) and WE43 (**right**) performed with a 10 and 100 mV/s scan rate in Ringer’s solution.

**Figure 4:**
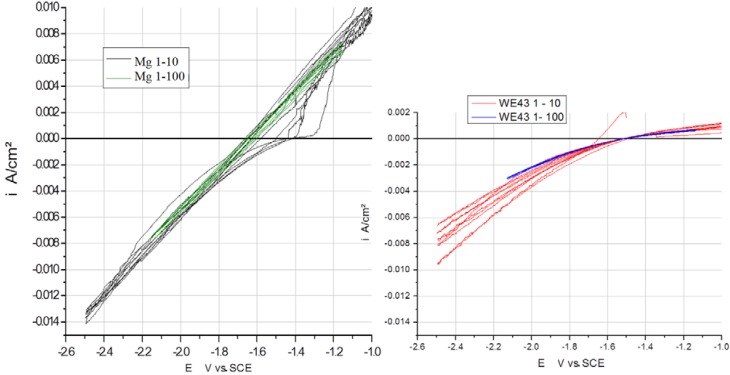
Linear, scaled presentation of cyclic *I* *vs.* *E* curves of pure Mg (**left**) and WE43 (**right**) performed with two different scan rates, 10 and 100 mV/s, in Ringer’s solution.

**Figure 5:**
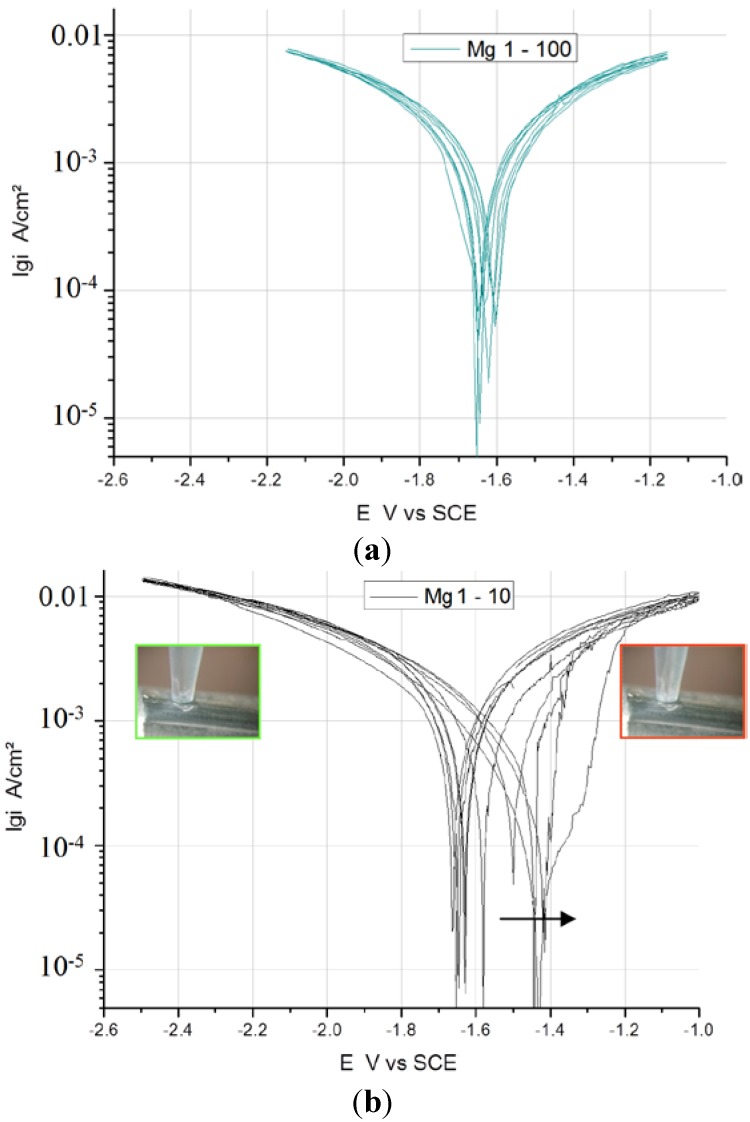
Detailed description of measurements on Mg at (**a**) 100 and (**b**) 10 mV/s.

**Figure 6:**
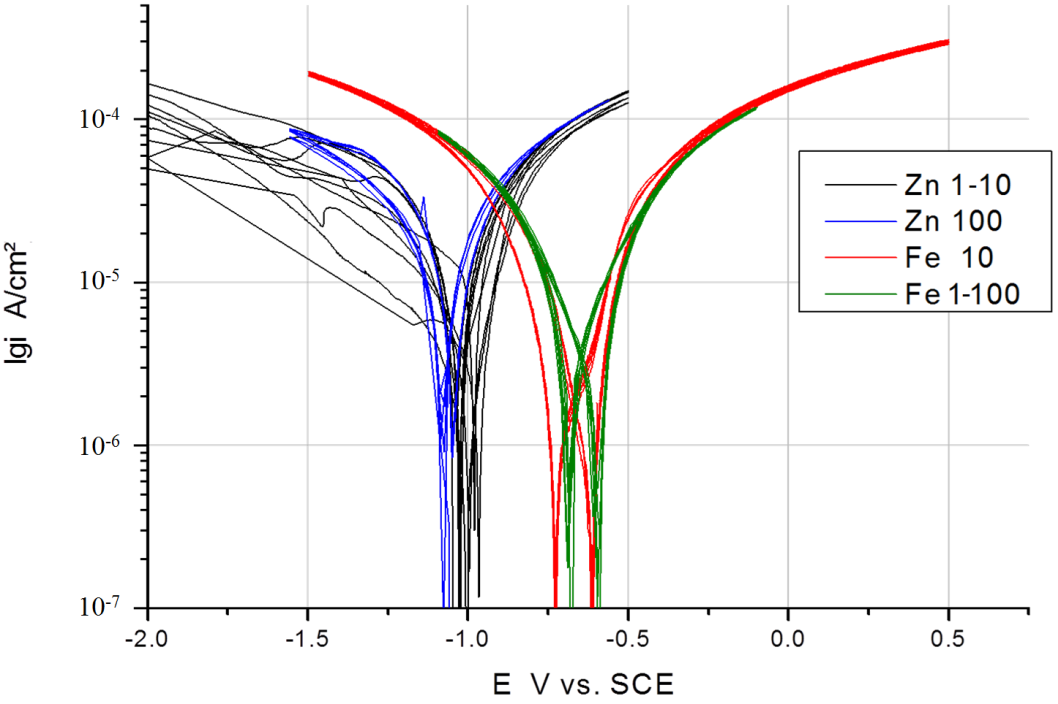
Comparison of *I* *vs.* *E* curves of Zn and Fe performed with a 10 and 100 mV/s scan rate in Ringer’s solution (*E*_Zn_ = −1.004 V *vs.* SCE; *E*_Fe_ = −0.685 V *vs.* SCE).

In Figure 3, Figure 4 and Figure 5, the measured voltammograms on Zn, Fe, Mg and WE43 are compared using a 10 and a 100 mV/s scan rate. It was expected that a higher scan rate would increase the corrosion current, due to the charging of the surface. However, only in the case of Fe can a shift be observed (Figure 6); in the cases of WE43 and Zn, a relatively good agreement between *I* *vs.* *E* curves performed with 10 and 100 mV/s can be analyzed. The differences between pure Mg and WE43 are clearly seen in Figure 3. The presentation of the same cyclic voltammograms in a linear scale, Figure 4, shows that the changes in the corrosion resistance were well reversible by the difference in the slope; thereby, the corrosion resistance of WE43 appears significantly higher. The curves of pure Mg differ significantly as a result of the scan variation using 10 and 100 mV/s. Figure 5 shows this in detail. The polarization curves at a scan rate of 100 mV/s have a reversible shape and a slight anodic shift of the corrosion potential of the forward scan. The polarization curves at a scan rate of 10 mV/s show differences for the forward scans (cathodic to anodic): the corrosion potential and breakdown potentials shift in the anodic direction from cycle to cycle with the increased extension of the “passive”-like range. However, in the anodic arm of the forward scan, hydrogen evolution takes place, and bubbles can be observed; see the red framed picture on the right in Figure 5. The 10 mV/s backward scans (anodic to cathodic) are all symmetrical and then reach the same performance as the 100 mV/s scans. In the cathodic part, no hydrogen evolution takes place and no bubbles appear; see the green framed picture on the left in Figure 5. That means that after polarization during the 10-mV/s forward scan, the breakdown of any type of passive layer on the surface of Mg has occurred. For the following 10-mV/s backward scan, the surface appeared free and to be directly acting without any neutralization of Mg ions by galvanic coupling. From this, it can be concluded that the whole surface and all Mg ions can be reduced during the backward scan. The calculated results of the voltammograms are collected in Table 2. The corrosion potentials lie in the range of the OCPs, and the assessed corrosion rates have similar values as found in the literature [1].

**Table 2:** Calculated mean values of the corrosion rates (CR) of Mg and WE43 based on *Ivs.E* curves with different scan rates in Ringer solution.

Material Scan Rate Cathodic Threshold Potential	*i*_corr_*^f^*^w^ (A/cm²)	CR *^f^*^w^ (mm/year)	*E*_corr_*^f^*^w^ (V*vs.* SCE)	*i*_corr_*^b^*^w^ (A/cm²)	CR *^b^*^w^ (mm/year)	*E*_corr_*^b^*^w^ (V *vs.* SCE)	*R*_p_ (Ω cm²)
WE43 10 mV/s E_T_ = −1 V *vs.* OCP	3.92 × 10^−5^	5.68 × 10^−1^	−1.51214	6.67 × 10^−5^	9.66 × 10^−1^	−1.5432	276
SD	7.71 × 10^−6^	1.12 × 10^−1^	0.01187468	6.47 × 10^−5^	9.37 × 10^−1^	0.05866018	
WE43 100 mV/s E_T_ = −0.5 V *vs.* OCP	2.75 × 10^−5^	3.98 × 10^−1^	−1.51482	2.48 × 10^−5^	3.59 × 10^−1^	−1.5146	393
SD	1.07 × 10^−6^	1.55 × 10^−2^	0.00584739	1.52 × 10^−6^	2.20 × 10^−2^	0.00335857	
WE43 100 mV/s E_T_ = −1 V *vs.* OCP	3.03 × 10^−5^	4.38 × 10^−1^	−1.54254	2.91 × 10^−5^	4.22 × 10^−1^	−1.5400	
SD	4.77 × 10^−6^	6.92 × 10^−2^	0.00609286	2.95 × 10^−6^	4.28 × 10^−2^	0.00758571	
Mg 10 mV/s E_T_ = −1 V *vs.* OCP	1.48 × 10^−4^	2.14	−1.4752	2.08 × 10^−4^	3.01	−1.6444	59
SD	9.34 × 10^−5^	1.35	0.0671203	8.39 × 10^−6^	1.22 × 10^−1^	0.01472678	
Mg 100 mV/s E_T_ = −0.5 V *vs.* OCP	1.68 × 10^−4^	2.44	−1.61504	1.72 × 10^−4^	2.49	−1.6475	68
SD	1.24 × 10^−5^	1.80 × 10^−1^	0.01142817	3.56 × 10^−6^	5.16 × 10^−2^	0.00302476	
Mg 100 mV/s E_T_ = −1 V *vs.* OCP	5.40 × 10^−5^	7.82 × 10^−1^	−1.5261	5.00 × 10^−5^	7.24 × 10^−1^	−1.5457	
SD	2.51 × 10^−6^	3.64 × 10^−2^	0.00435603	2.28 × 10^−6^	3.31 × 10^−2^	0.00314754	

*i*_corr_ = corrosion current density; *f*w = forward scan; CR = corrosion potential; *b*w = backward scan; *E*_corr_ = corrosion potential; *R*_p_ = polarization resistance; *E*_T_ = cathodic threshold potential; SD = standard deviation.

## 4. Discussion

In Table 3 and Table 4, the OCPs of various Mg and Mg alloys in various electrolytes found in the literature are listed. Considering these data from the literature and the data from our own experiments on Mg and WE43 (Table 1), it is clear that the OCP values are anodically far away from the standard potential of magnesium, which is, according to Perrault [26], *vs.* normal hydrogen electrode (NHE) as follows:

*E*_0_ = −2.363 V (*vs.* NHE) or −2.604 V (*vs.* SCE) (7)

The standard potential would be expected if the potential is related to Equation (8), and a concentration of Mg^2+^ of 1 mol/L is liberated in the vicinity of the surface of the working electrode, in addition, without any coupled reaction, for example Equations (11) and (12).

*Mg* ⇔ *Mg*^2+^ + 2*e*^−^ (8)

However, the measured OCP values are closer to the standard potential for MgH_2_, which are:

*E*_0_ = −1.114 V (*vs.* NHE) for Equation (11) (9)

*E*_0_ = −1.256 V (*vs.* NHE) for Equation (12) (10)

*Mg*^2+^ + 2*H*^+^ + 4*e*^−^ ⇔ *MgH*_2_ (11)

*Mg(OH)*_2_ + 2*H*^+^ + 4*e*^−^ ⇔ *MgH*_2_ + 2*OH*^−^ (12)

Considering the thermodynamic data [27], it is obvious that MgH_2_ has a higher stability as Mg(OH)_2_. The hydride is prominent in the presence of Cl^−^ ions in the physiological environment, showing a much higher concentration than OH^−^ ions at pH 7. Based on that, it can be assumed that as the first step, a MgH_2_ film is created on the Mg surface, proposed also by Perrault [27]. That is also supported by the detection of the Mg hydride in the surface film on magnesium by immersion for 2 min [19]. Moreover, other facts support the hypothesis of a stable intermediate state of MgH_2_. On one side, the liberation of hydrogen from MgH_2_ needs additional energy, which will delay the activation. On the other side, for the formation of MgH_2_, a high amount of hydrogen is necessary, which can be seen from the phase diagram Mg–H [28], and the high amount is available due to Equations (11) and (12).

**Table 3:** Mg in various electrolytes.

Electrolyte	pH	OCP *vs.* NHE	Reference
0.05 M NaOH, 0.186 M H_3_BO_3_, 0.0002 M NaCl	8.9	−1.48	p. 18 in Perrault 26
0.1 M NaCl	10.6	−1.44	p. 18 in Perrault 26
0.2 N MgCl_2_ 8.33 × 10^−3^ N HCl	1.85	−1.72	p. 19 in Perrault 26
1 N NaCl	7	−1.72	p. 20 in Perrault 26
1 N Na_2_SO_4_	7	−1.75	p. 20 in Perrault 26
1 N HCl	1	−1.49	p. 20 in Perrault 26
3% NaCl		−1.39 → −1.44	Volovitch *et al.* [29]
Simulated body fluid (SBF)	7	−1.51	Gao *et al.* [30]
5% NaCl	7	−1.34 → −1.51	Chong *et al.* [31]
3.5% NaCl	7	−1.36 → −1.62	Shin *et al.* [32]
0.1 N NaCl	7	−1.66	Zhao *et al.* [33]

**Table 4:** Mg–Al alloys and Mg–Al–Zn alloys.

Mg-Alloy	Electrolyte	pH	OCP *vs.* SCE	Reference
Mg–1Al	3.5% NaCl	7	−1.62	Lee *et al.* [23]
Mg–3Al	3.5% NaCl	7	−1.62	Lee *et al.* [23]
Mg–6Al	3.5% NaCl	7	−1.64	Lee *et al.* [23]
Mg–9Al	3.5% NaCl	7	−1.65	Lee *et al.* [23]
Mg–12Al	3.5% NaCl	7	−1.63	Lee *et al.* [23]
Mg–12Al–1Zn	3.5% NaCl	7	−1.58 → −1.64	Lee *et al.* [23]
AZ91	Simulated body fluid (SBF)	7	−1.4 3 → −1.48	Kannan *et al.* [34]
AZ91	5% NaCl	7	−1.55	Chong *et al.* [31]
AZ80	5% NaCl	7	−1.55	Chong *et al.* [31]
AZ65	5% NaCl	7	−1.58	Chong *et al.* [31]

Having the thermodynamic considerations as a base, the key question of the discussion is how to interpret the kinetics of a cyclic voltammogram on Mg in a chloride containing solution. First, the polarization curves at a scan rate of 100 mV/s are considered. They have a reversible shape and a very slight anodic shift of the corrosion potential of the forward scan. The forward scans at scan rates of 10 mV/s are different: the corrosion potentials, as well as the breakdown potentials shift in the anodic direction from cycle to cycle with the increased extension of the “passive”-like range. Independently of the scan rate, in all anodic arms of the forward scan toward the anodic threshold, a strong hydrogen evolution and the take-off of bubbles took place, for pure Mg and also for WE43. That means that a breakdown of any partially protective film occurred.

The backward scans are very symmetric for both scan rates and show the same corrosion potential of about −1.65 V, and no bubble take-off took place. After reaching a breakdown during the end of the forward scan, the following backward scan may start on an electrochemically active and free surface. During the entire backward scan, the whole surface and all Mg ions could then be reduced. Therefore, the backward scan might be used for a more reliable calculation of the corrosion rate for Mg and Mg alloys. The calculation of the corrosion rate appears to be correct, as long as the electron flow goes through the external circuit. Comparing the results of scan rates of 100 mV/s with measurements using the well-accepted scan rate of 10 mV/s by application of the mini cell system (MCS), the scan rate of 100 mV/s appeared to be appropriate to measure the electron flow without perturbation by any side reaction, although 100 mV/s is very high. However, the applied method using scan rates of 100 mV/s to assess the degradation or corrosion of Mg and Mg alloys can only be valid for very small contact areas, as realized with MCS.

Based on the discussion above, the following mechanism can be proposed for the Mg reaction pathway during electrochemical measurements and be applied to interpret our results.

(1) Initial step: As Perrault [26] described, first, MgH_2_ film will be created on the Mg or Mg alloy surface, according to Equations (13) and (14). These reactions cause an increase of pH in the vicinity of the surface, because the protons from the water equilibrium are consumed during the reaction.
  *Mg* ⇒ *Mg*^2+^ + 2*e*^−^ (13)
  *Mg*^2+^ + 4*e*^−^ + 2*H*^+^ ⇒ *MgH*_2_ (14)
(2) Next step: Under stationary conditions as a consequence of the increase of OH^−^ ions at the surface, a chemically degradation of the MgH_2_ layer takes place with the liberation of 1 mol of hydrogen at the end; see Equations (15) and (16). The reaction pathway in Equation (6) would explain that Mg(OH)_2_ or MgO under vacuum conditions can be detected [18].
  *MgH*_2_ + 2*OH*^−^ ⇒ *Mg(OH)*_2_ + 2*H*^+^ + 4*e*^−^ (15)
  *MgH*_2_ + 2*OH*^−^ ⇒ *Mg(OH)*_2_ + *H*_2_ + 2*e*^−^ (16)
(3) OCP: At the OCP, measured in the range between −1.4 to −1.7 V (*vs*. SCE), reactions according to Equations (11) and (12) take place.
(4) Polarization: During the polarization, the cathodic and the anodic arm of the *I*–*E* curve can be discussed separately. The cathodic arm follows Equations (11) and (12). In the cathodic arm of the forward scan, the formation of the MgH_2_ film takes place. This film induces in the anodic arm of the forward scan a so-called passivation effect, because of the temporary stability of the MgH_2_ and delayed H_2_ formation following Equation (15) or (16). That is the reason that H_2_ formation takes place in the anodic arm of the forward scan. The Mg oxidation becomes dominant; no passivation occurs, and the NDE can be explained considering that a part of the surface will be changed chemically according to Equation (6). Further discussion has to be separated for the different scan rates of 10 and 100 mV/s.

### 4.1. Mixed Potential Effect during Polarization with a Scan Rate of 10 mV/s

**Figure 7:**
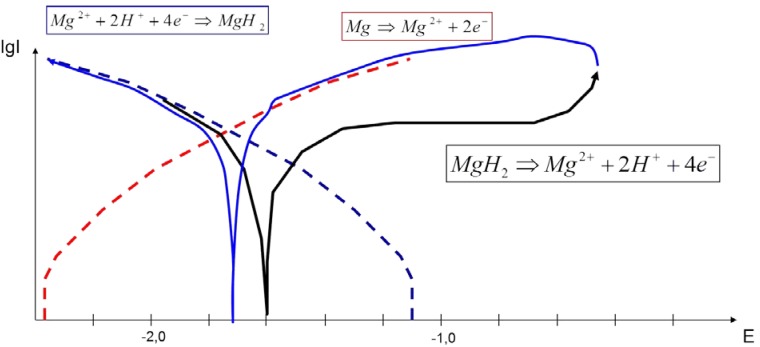
The scheme to describe the mixed potential effect on Mg or Mg alloy surfaces for an interpretation of the shape of *I vs.* *E* curves using a slow scan rate of 10 mV/s. Dashed lines = the anodic part of Mg oxidation (red) and the cathodic part of the hydrogen evolution (blue); Full line = the forward scan (black) and the backward scan (blue).

From the measured voltammograms, it was found that the backward scans generate higher current densities, as well as higher corrosion current densities than the forward scans, which can be explained by the fact that there is no protecting hydride film, and finally, the corrosion potential of the backward scan lies closer to the Mg standard potential; see Figure 7. The *I*–*E* curves of the studies of Atrens *et al.* [2] performed with a very slow scan rate of 1 mV/s show a sharp slope, more of a kind of switch to high current densities after the step over the corrosion potential in the anodic direction. This can be explained by the superposition of two processes, which deliver electrons in the measurement circuit according to the following equations:

*Mg* ⇒ *Mg*^2+^ + 2*e*^−^ (17)

*MgH*_2_ ⇒ *Mg*^2+^ + 2*H*^+^ + 4*e*^−^ (18)

### 4.2. Passive-Like Behavior during Polarization with a Scan Rate of 100 mV/s

Under various scan rates, a more or less passive-like behavior can be observed. Two different pathways can be discussed. One possibility is the direct conversion of MgH_2_ by anodic polarization following Equation (18). The second pathway is shown in Equations (19) and (20), which are more complex, as hydrogen will be liberated by an attack of OH^−^ ions at the surface, as well as electrons, causing a slight increase of the current in the circuit.

*MgH*_2_ + 2*OH*^−^ ⇒ *Mg(OH)*_2_ + *H*_2_ + 2*e*^−^ (19)

*MgH*_2_ + 2*Cl*^−^ ⇒ *MgCl*_2_ + *H*_2_ + 2*e*^−^ (20)

This is in agreement with Swiatowska *et al.* [35], who could show that the reaction order of Mg is *z* = 2. The creation of a surface layer and the influence of the composition of the electrolyte in the vicinity of the surface as key factors for the corrosion rate were described by Volovitch *et al.* [29]. Based on that, it can be concluded that in the case of “steady-state” electrochemical measurements, the application of the Tafel method for the assessment of corrosion rates is not possible. In the case of faster scan rates, an application of the polarization resistance technique for the calculation of the exchange current density seems to be possible, as long as the *I*–*E* curves show a passive behavior without the hydrogen evolution at the point of the calculation of the degradation/corrosion of Mg and Mg alloys, which are given in the vicinity of *E*_corr_ for the backward scans. This can be also a method for a reliable prediction of the degradation of a Mg-based biomaterial *in vitro*, which was mentioned as a problem [16]. A similar approach would be described by Bender *et al.* [17], where the existence of the Mg^+^ intermediate was denied.

### 4.3. Development of New Mg Alloys

**Figure 8:**
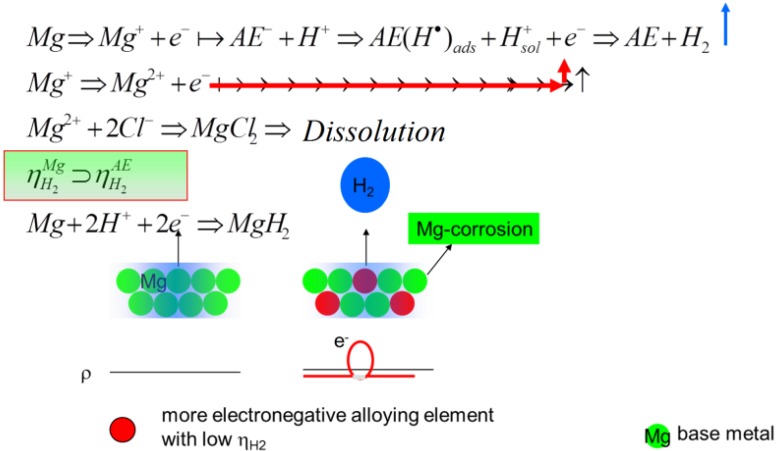
The scheme to describe the effect of the electronegative alloying elements in dependence of the study of Ezaki *et al.* [36].

For new biomaterials based on Mg, it can be proposed to prepare microstructures having a distribution of alloying elements in a way that the hydrogen evolution can spread over the whole surface by the galvanic coupling effect, whereby the elements with a low hydrogen overpotential act as a cathode in that couple. Ezaki *et al.* [36] could show that by the mixing of more electronegative elements into the base metal, the HER can be driven by its distribution along the surface, as well as into the volume; see Figure 8. This could be proven by Tie *et al.* [37] by adding Ag to Mg and by Zhang *et al.* [38] using a grain refinement up to nano-crystallinity. If it is possible to avoid pitting corrosion, the NDE will disappear, as Drazic *et al.* [39] found for aluminum. At least combining the ideas, like grain refinement, and adding electronegative alloy elements in a way that a more or less homogeneous distribution of elements along the surface and in the bulk can be realized, no NDE should appear during the electrochemical measurements and a more homogeneous degradation should be given, which is one of the goals for the manufacturing of degradable metallic biomaterial devices.

## 5. Materials and Methods

For the electrochemical measurements, 99.98% pure elements, Zn, Fe, Al (Good Fellow, Bad Nauheim, Germany), Mg ≥ 99.9% (Sigma-Aldrich, München, Germany) and WE43 were used. WE43 (Magnesium Elektron, Swinton, UK) is an Mg alloy consisting of Mg, 3.7%–4.3% Y, 2.4%–4.4% rare earth elements (RE) and, of that, 2% Nd and 0.4% Zr. Ringer’s solution (Fresenius Kabi, Bad Homburg, Germany), composed of 9 g NaCl, 0.24 g CaCl_2_·6H_2_O, 0.43 g KCl, 0.2 g NaHCO_3_ in 1000 mL distilled water, was used as the electrolyte for the measurements. Before measurement, the specimens were ground and polished with SiC paper up to grit 2500 and cleaned with ethanol 96%.

**Figure 9:**
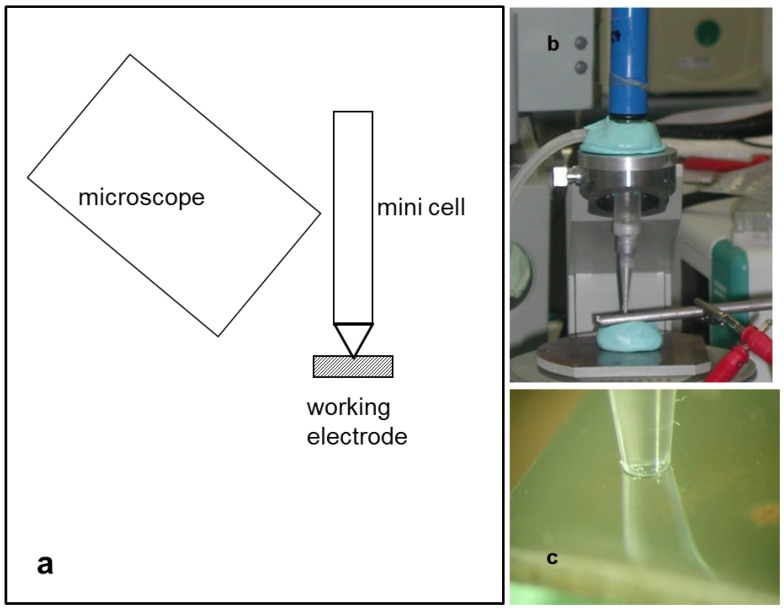
Setup of the measurement: (**a**) overview scheme; (**b**) mini-cell-system (MCS); and (**c**) stereo microscopic view of the measurement area.

All electrochemical measurements were performed with the mini-cell-system (MCS), a three-electrode system with a measurement area of 0.008 cm², and SCE as the reference electrode. The setup is shown in Figure 9, where a stereo microscope was adapted to observe the hydrogen evolution during open circuit potential (OCP) measurement and polarization. OCP measurements for 15 min followed by cyclic voltammetry with three different cathodic threshold potentials were performed. The different threshold potentials were calculated from the OCP, less 0.5, 1.0 and 1.5 V in the cathodic direction. The anodic threshold potential of the polarization curve for all scans was at 0.5 V anodic from OCP. Two scan rates, 100 and 10 mV/s, and 5 cycles for each measurement per threshold were applied.

## 6. Conclusions

The focus of this study was a new approach for the interpretation of electrochemical measurements, as well as the generation of a new electrochemical method by the use of rapid scans of 100 mV/s. The use of backward scans showed the possibility of more reliable calculation of corrosion rates combined with the application of MCS.
